# Exogenous glutathione reverses meropenem resistance in carbapenem-resistant *Klebsiella pneumoniae*

**DOI:** 10.3389/fphar.2023.1327230

**Published:** 2023-12-19

**Authors:** Juan Yi, Chao Liu, Ping Yang, Zhen-chao Wu, Chun-jing Du, Ning Shen

**Affiliations:** ^1^ Institute of Medical Technology, Peking University Health Science Center, Beijing, China; ^2^ Department of Pulmonary and Critical Care Medicine, Peking University Third Hospital, Beijing, China; ^3^ Department of Infectious Disease, Peking University Third Hospital, Beijing, China; ^4^ Center of Infectious Disease, Peking University Third Hospital, Beijing, China

**Keywords:** glutathione, synergy, meropenem, carbapenem-resistant *Klebsiella pneumoniae*, antimicrobial resistance, glycerophospholipid metabolism

## Abstract

**Background:** The rate of carbapenem-resistant *Klebsiella pneumoniae* (CRKP) infection has been increasing rapidly worldwide and, poses a significant risk to human health. Effective methods are urgently needed to address treatment failures related to antibiotic resistance. Recent research has reported that some drugs in combination with antibiotics have displayed synergistic killing of resistant bacteria. Here, we investigated whether glutathione (GSH) can synergize with meropenem, and enhance its effectiveness against CRKP.

**Methods:** Synergistic activity was assessed by checkerboard and time-killing assays. The mechanism of these combinations was assessed by total ROS and membrane permeability assays. The bacterial metabolites were assessed by LC‒MS/MS.

**Results:** The FICIs of GSH and meropenem were approximately 0.5 and the combined treatment with GSH and meropenem resulted in a more than 2log10 CFU/mL reduction in bacteria compared to the individual treatments. These findings indicated the synergistic effect of the two drugs. Moreover, the meropenem MIC of CRKP was reduced to less than 4 mg/L when combined with 6 mg/mL GSH, indicating that GSH could significantly reverse resistance to meropenem in bacteria. The production of ROS in bacteria was determined by flow cytometry. After adding GSH, the ROS in the GSH group and the combined group was significantly higher than that in the control and meropenem groups, but there was no significant difference between the combined and GSH groups. The metabolic disturbance caused by GSH alone and in combination with meropenem was significant intracellularly and extracellularly, especially in terms of glycerophospholipid metabolism, indicating that the synergistic effect of the combined use of GSH and meropenem was relevant to glycerophospholipid metabolism. In addition, we measured the cell membrane permeability. The cell membrane permeability of the combination group was significantly higher than that of the blank control or monotreatment groups. This confirmed that the GSH can serve as a meropenem enhancers by disturbing glycerophospholipid metabolism and increasing cell membrane permeability.

**Conclusion:** GSH and meropenem display a synergistic effect, wherein GSH increases the sensitivity of CRKP to meropenem. The synergy and susceptibility effects are thought to related to the increased membrane permeability resulting from the perturbations in glycerophospholipid metabolism, presenting a novel avenue for CRKP treatment.

## 1 Introduction

*Klebsiella pneumoniae* is an opportunistic Gram-negative pathogen that frequently causes nosocomial infections in humans and accounts for a significant proportion of hospital-acquired urinary tract infections, pneumonia, and bloodstream infections (Pendleton et al., 2013). In addition to its importance as a nosocomial pathogen, *Klebsiella pneumoniae* is notorious for its high frequency and diversity of antimicrobial resistance (AMR) genes. In recent years, there has been an increasing global incidence of *K. pneumoniae* strains with multidrug resistance traits. Carbapenems are considered the preferred antibiotics to treat health care-associated infections caused by ESBL and AmpC-producing Enterobacteriaceae, particularly *Klebsiella pneumonia*. However, in recent years, carbapenem-resistant Enterobacteriaceae (CRE) has been noted as one of the critical-priority bacteria by the WHO (Tacconelli et al., 2018; Zhen et al., 2021). In particular, carbapenem-resistant *K. pneumonia* (CRKP) accounted for a large proportion of CRE cases, according to a report from the China CRE Network (Zhang et al., 2018). In addition, CRKP usually exhibits multidrug resistance against most β-lactams, fluoroquinolones and aminoglycoside antibiotics, which complicates the selection of adequate treatments and contributes to overall mortality; this presents a challenge because antimicrobial treatment options remain very restricted.

As efforts to develop antimicrobial agents that can effectively kill MDR bacteria have not been successful thus far, there is an urgent need for drugs that can kill or prevent infection by these bacteria. Recent research has reported that some drugs in combination with antibiotics have displayed synergistic killing against resistant bacteria (Copp et al., 2020; Kim et al., 2020; Liu et al., 2021; Liu et al., 2022). Previous studies revealed that glutamine promoted antibiotic uptake to increase the killing of MDR bacteria, myo-inositol improved the host’s ability to eliminate balofloxacin-resistant *Escherichia coli*, and L-alanine metabolism conferred aminoglycoside resistance via Na^+^-NQR (Chen et al., 2015; Jiang et al., 2020; Zhao et al., 2021). These studies revealed the effect of the combination of nonantibacterial substances and antibiotics on the treatment of drug-resistant bacteria and provided a new direction for the treatment of drug-resistant bacteria.

Glutathione (GSH) is an antioxidant synthesized in most Gram-negative bacteria and a few Gram-positive bacteria (Smirnova and Oktyabrsky, 2005). In addition to its role in maintaining the particular reduction potential, GSH is also related to antibacterial activity and biofilm clearance (Klare et al., 2016). Das et al. reported that the growth and biofilm activity of all bacteria decreased by more than 50% under 30 mM exogenous GSH, whereas the effects on different bacteria were different (Das et al., 2019). In addition, exogenous GSH mediated ciprofloxacin protection in *Escherichia coli* by neutralizing antibiotic-induced oxidative stress, increasing the efflux of antibiotics, and promoting acid shock survival (Goswami and Narayana Rao, 2018). GSH-mediated protection has been observed when used with quinolones and aminoglycosides, such as ciprofloxacin, norfloxacin and spectinomycin (Goswami et al., 2006; Goswami and Narayana Rao, 2018). According to previous studies (Klare et al., 2016; Alharbe et al., 2017; Das et al., 2019), GSH differentially modulates the antibacterial activity of diverse antibiotics in various kinds of bacteria.

However, it is not known whether GSH can restore the activity of meropenem against CRKP. In this study, meropenem in combination with GSH was evaluated against clinical CRKP isolates. In addition, we explored whether the mechanism by which meropenem synergizes with GSH stems from elevated membrane permeability resulting from perturbations in glycerophospholipid metabolism.

## 2 Methods

### 2.1 Bacterial strains

This study included 30 CRKP strains isolated from the samples of 30 individual patients. These patients were enrolled from a retrospective cohort study in a tertiary care hospital. All specimens were obtained between February 2020 and February 2021. The isolates were confirmed by the combination of mass spectrometry and the Vitek 2 system (bioMérieux, Marcy-l’Étoile, France). The definition of CR was the resistance of the strains to meropenem or imipenem in antibiotic susceptibility testing (AST) (Magiorakos et al., 2012). The clinical information of these patients was collected, including age, sex, admitted department, isolation time, specimen type, and outcome in 30 days. The study was approved by the Peking University Third Hospital Medical Science Research Ethics Committee (M2021545).

### 2.2 Antibiotic susceptibility testing

The ASTs of these isolates was conducted by the Vitek 2 system and included the following agents: meropenem, imipenem, ceftazidime, cefepime, piperacillin/tazobactam, cefoperazone/sulbactam, levofloxacin, amikacin, minocycline, and trimethoprim/sulfamethoxazole. The AST results were interpreted according to the Clinical and Laboratory Standards Institute (CLSI) guidelines. Furthermore, the minimum inhibitory concentrations (MICs) of GSH and meropenem were determined by the microbroth dilution method as previously described (Timu et al., 2006). GSH and meropenem were prepared in CAMH broth. A final bacterial suspension of 5 × 10^5^ CFU/mL was added to each well, and the bacteria were incubated with diluted broth for 18 h at 37°C. The MIC was the lowest concentration that inhibited the visible growth of the bacteria.

### 2.3 Checkerboard assays

The synergy between meropenem and GSH was tested using the checkerboard method (Flamm et al., 2019). Meropenem and GSH were prepared with twofold serial dilutions and mixed to create different combinations of concentrations. The concentrations employed in the checkerboard assays were established based on the MIC of each agent that was previously determined for each specific isolate. A final bacterial suspension of 5 × 10^5^ CFU/mL was added to each well. After incubation for 18 h at 37°C, the fractional inhibitory concentration was calculated as the MIC of the combination divided by that of each compound alone. The sum of the fractional inhibitory concentrations of the two drugs was denoted as the fractional inhibitory concentration index (FICI). The results were interpreted as synergistic (FICI ≤0.5), additive (0.5 < FICI ≤ 1), or indifferent (FICI >1) (Hall et al., 1983; Antonelli et al., 2022).

### 2.4 Time-killing kinetics assays

A time-killing assay was performed to determine the synergy between GSH and meropenem as described by White et al. (1996) with few modifications. Briefly, overnight cultured bacteria were suspended and diluted to 1 × 10^6^ CFU/mL in CAMH broth. Diluted bacteria were then incubated with GSH (6 mg/mL), meropenem (4 mg/L), and the combination. Aliquots (100 μL) were taken out at 0, 4, 8, 12 and 24 h postinoculation and serially diluted in PBS solution to determine viable counts. Diluted samples (100 μL) were uniformly plated on MH agar plates. After culturing at 37°C for 18 h, the total bacterial counts (CFU/mL) were recorded. Synergy was defined as a ≥2 log10 CFU/mL reduction at 24 h in a combination when compared to the CFU/mL of the most active individual drug (Gómara and Ramón-García, 2019).

### 2.5 Total ROS assay

2′,7′-dichlorofluorescein diacetate (DCFH-DA) dye was used to measure reactive oxygen species (ROS) levels in CRKP as previously described (Yu and Guo, 2022). In brief, each strain was grown overnight in MH agar at 37°C. A single colony of each strain was inoculated into 5 mL of LB medium for 6–8 h with shaking at 37°C and 200 rpm. The bacteria were collected by 4,000 × g centrifugation, washed with PBS twice, and resuspended to 0.5 MCF. Briefly, the suspension was treated with 4 mg/L meropenem, 6 mg/mL GSH or the combination at 37°C for 2 h. Then, bacteria collected by 4,000 × g centrifugation were washed twice with PBS, followed by the addition of DCFH-DA (10 μM) and incubation in the dark at 37°C for 30 min. After loading the dye, the probe was washed twice with PBS, and the fluorescence intensity was measured by flow cytometry within half an hour.

### 2.6 Bacterial metabolite extraction

Metabolite extraction was performed as described previously with a few modifications (Peng et al., 2015; Ye et al., 2021). Isolates cultured overnight were inoculated into LB broth at 37°C and incubated at 200 r/min for 6–8 h. The suspension was centrifuged and treated with 4 mg/L meropenem, 6 mg/mL GSH or the combination at 37°C for 4 h. And the bacteria cultured in broth for 4 h without any agent treatment served as a blank control group. The culture was diluted to the same concentration with fresh medium and centrifuged at 5,000 × g for 20 min at 4°C to separate bacteria from the supernatant. Bacteria were then washed twice in PBS at 4°C, and the supernatants were filtered through a 0.2 μm membrane filter. The bacteria and medium were both stored at −80°C until metabolites were extracted with cold methanol. When extracting, equivalent cells or medium were quenched using methanol (containing isotopically-labeled internal standard mixture) at −40°C and collected by centrifugation at 13,800 × g for 15 min. The resulting supernatant was transferred to a fresh glass vial for LC‒MS analysis.

### 2.7 LC‒MS/MS analysis and data preprocessing

A UHPLC system (Vanquish, Thermo Fisher Scientific) with a UPLC HSS T3 column coupled to an Orbitrap Exploris 120 mass spectrometer (Orbitrap MS, Thermo) was employed to perform LC‒MS/MS analysis. The mobile phase consisted of 5 mmol/L ammonium acetate, 5 mmol/L acetic acid in water (A) and acetonitrile (B). The information-dependent acquisition (IDA) mode was applied to acquire MS/MS spectra by acquisition software (Xcalibur, Thermo). The raw data were converted to the mzXML format by ProteoWizard and processed with XCMS (Sm et al., 2006) for peak detection, extraction, alignment, and integration. Then, an MS2 database (BiotreeDB) was applied for metabolite annotation. The cutoff for annotation was set at 0.3.

### 2.8 Membrane permeability assay

The treatment of bacteria for this assay was the same as the method for the ROS assay. The membrane permeability of the treated bacteria was measured based on the fluorescence intensity of propidium iodide (PI)-labeling. PI (10 μM) was added to the washed bacterial precipitate and the sample was incubated for 30 min at 37°C after shaking and mixing. Finally, the fluorescence intensity of each sample was detected by flow cytometry.

### 2.9 Statistical analysis

Statistical analysis was carried out by GraphPad Prism 8.3.5 and SIMCA V16.0.2. Except the data of metabolomic, all data were presented as the mean ± standard deviation. Student’s t-test or one-way ANOVA were used to determine the differences between groups and the differences were significant when *p*-value is less than 0.05 (*p < 0.05, **p < 0.01, ***p < 0.001). Metabolomic was analyzed by Student’s t-test, principal component analysis (PCA) and orthogonal projections to latent structures-discriminant analysis (OPLS-DA). The metabolites of the GSH, meropenem or the combination group were compared to the blank control group. The cutoff of the differentially expressed metabolites was based on the following two indicators: the *p*-value of Student’s t-test was less than 0.05, and the variable importance in the projection (VIP) of the OPLS-DA model was greater than 1. Taking *Klebsiella pneumoniae subsp. pneumoniae* MGH 78578 (serotype K52) as a reference, the screened differentially abundant metabolites were mapped using the KEGG pathway database (http://www.kegg.jp/kegg/pathway.html).

## 3 Results

### 3.1 Antimicrobial susceptibility and clinical characteristics of the *Klebsiella pneumoniae* isolates

The antimicrobial susceptibility results for each isolate are displayed in Table 1. All 30 isolates were resistant to meropenem (MIC, 32–128 mg/L), imipenem (MIC, ≥16 mg/L), cefepime (MIC, ≥32 mg/L), ceftazidime (MIC, 16 to ≥64 mg/L), cefoperazone-sulbactam (MIC, ≥64 mg/L), piperacillin-tazobactam (MIC, ≥128 mg/L), and levofloxacin (MIC, ≥8 mg/L). Most isolates were resistant to amikacin (73.3%; MIC, 2 to ≥16 mg/L). Approximately half of the 30 isolates were resistant to trimethoprim-sulfamethoxazole (56.7%). The lowest resistance rate was observed for minocycline (40.0%). According to the AST results, all isolates had CR phenotypes. These strains were mostly isolated from sputum specimens, primarily originating from the ICU, emergency department, and geriatric wards; these isolates were associated with a 50% mortality rate within 30 days for patients (Table 2).

**TABLE 1:** Antimicrobial susceptibility test of the enrolled isolates.

Isolate	MEM	IPM	FEP	CAZ	CSL	TZP	MNO	AMK	LVX	SXT	GSH
R1	≥16	≥16	≥32	≥64	≥64	≥128	8	4	≥8	≥320	6
R2	≥16	≥16	≥32	≥64	≥64	≥128	≥16	≥64	≥8	≥320	12
R3	≥16	≥16	≥32	≥64	≥64	≥128	≥16	≥64	≥8	≥320	6
R4	≥16	≥16	≥32	≥64	≥64	≥128	≥16	≥64	≥8	≥320	12
R5	≥16	≥16	≥32	≥64	≥64	≥128	4	≥64	≥8	≥320	12
R6	≥16	≥16	≥32	≥64	≥64	≥128	4	≥64	≥8	≥320	12
R7	≥16	≥16	≥32	≥64	≥64	≥128	8	≥64	≥8	≥320	12
R8	≥16	≥16	≥32	≥64	≥64	≥128	8	≤2	≥8	≥320	6
R9	≥16	≥16	≥32	≥64	≥64	≥128	≥16	≥64	≥8	≥320	12
R10	≥16	≥16	≥32	≥64	≥64	≥128	≥16	8	≥8	40	12
R11	≥16	≥16	≥32	≥64	≥64	≥128	≥16	≥64	≥8	≥320	12
R12	≥16	≥16	≥32	≥64	≥64	≥128	8	≥64	≥8	≥320	12
R13	≥16	≥16	≥32	32	≥64	≥128	2	≤2	≥8	2	12
R14	≥16	≥16	≥32	≥64	≥64	≥128	≥16	≤2	≥8	≤20	12
R15	≥16	≥16	≥32	≥64	≥64	≥128	≥16	≥64	≥8	≤20	12
R16	≥16	≥16	≥32	≥64	≥64	≥128	8	≥64	≥8	≥320	12
R17	≥16	≥16	≥32	≥64	≥64	≥128	≥16	≥64	≥8	≥320	12
R18	≥16	≥16	≥32	≥64	≥64	≥128	2	≥64	≥8	≤20	12
R19	≥16	≥16	≥32	≥64	≥64	≥128	4	≥64	≥8	40	12
R20	≥16	≥16	≥32	≥64	≥64	≥128	8	≤2	≥8	≤20	12
R21	≥16	≥16	≥32	≥64	≥64	≥128	2	≥64	≥8	160	12
R22	≥16	≥16	≥32	≥64	≥64	≥128	8	≥64	≥8	≤20	12
R23	≥16	≥16	≥32	≥64	≥64	≥128	≥16	≥64	≥8	≤20	12
R24	≥16	≥16	≥32	≥64	≥64	≥128	≥16	≥64	≥8	≤20	12
R25	≥16	≥16	≥32	≥64	≥64	≥128	8	≥64	≥8	≤20	12
R26	≥16	≥16	≥32	≥64	≥64	≥128	4	≥64	≥8	≤20	12
R27	≥16	≥16	≥32	≥64	≥64	≥128	4	≥64	≥8	≤20	6
R28	≥16	≥16	≥32	≥64	≥64	≥128	≥16	16	≥8	≥320	12
R29	≥16	≥16	≥32	≥64	≥64	≥128	4	≥64	≥8	≤20	12
R30	≥16	≥16	≥32	≥64	≥64	≥128	8	8	≥8	≤20	12

The MIC of various drugs, in mg/L (GSH, mg/mL). MEM, meropenem; IPM, imipenem; FEP, cefepime; CAZ, ceftazidime; CSL, cefoperazone-sulbactam; TZP, piperacillin-tazobactam; MNO, minocycline; AMK, amikacin; LVX, levofloxacin; SXY, trimethoprim-sulfamethoxazole; GSH, glutathione.

**TABLE 2:** Clinical characteristics of the enrolled isolates.

Isolate	Collection date	Specimen	Patient	Department	Sex	Age	Outcome in 30 days
R1	2021/1/12	sputum	P1	ICU	m	72	Survive
R2	2020/11/26	sputum	P2	Geriatric Ward	m	90	Death
R3	2020/9/26	sputum	P3	Emergency	f	87	Death
R4	2020/12/2	sputum	P4	ICU	m	68	Survive
R5	2020/3/12	sputum	P5	Geriatric Ward	f	85	Survive
R6	2020/2/3	sputum	P6	Geriatric Ward	m	84	Survive
R7	2020/9/8	blood	P7	ICU	m	75	Death
R8	2020/11/24	sputum	P8	ICU	f	87	Death
R9	2020/3/22	urine	P9	Emergency	f	57	Survive
R10	2020/10/20	urine	P10	Geriatric Ward	m	90	Survive
R11	2020/11/6	sputum	P11	ICU	f	67	Death
R12	2020/9/28	sputum	P12	ICU	f	83	Death
R13	2020/12/3	sputum	P13	Emergency	m	83	Death
R14	2021/2/1	sputum	P14	ICU	f	84	Death
R15	2020/12/7	blood	P15	ICU	m	66	Death
R16	2020/10/21	sputum	P16	Emergency	m	65	Survive
R17	2020/11/11	sputum	P17	Emergency	f	89	Death
R18	2020/11/4	sputum	P18	ICU	m	85	Survive
R19	2021/2/24	sputum	P19	Geriatric Ward	m	83	Survive
R20	2020/12/24	urine	P20	General surgery department	f	49	Survive
R21	2020/10/16	urine	P21	Geriatric Ward	f	83	Survive
R22	2020/10/15	urine	P22	ICU	m	38	Survive
R23	2020/11/27	sputum	P23	Emergency	f	86	Death
R24	2020/10/30	Broncho-alveolar lavage	P24	ICU	m	84	Death
R25	2020/10/5	sputum	P25	ICU	f	90	Survive
R26	2020/7/23	sputum	P26	Emergency	m	62	Survive
R27	2020/8/3	sputum	P27	Geriatric Ward	m	94	Death
R28	2021/1/4	sputum	P28	Emergency	f	86	Survive
R29	2020/8/12	secretion	P29	ICU	m	95	Death
R30	2021/1/16	sputum	P30	Emergency	m	82	Death

### 3.2 Synergistic effect of glutathione and meropenem against CRKP

**FIGURE 1:**
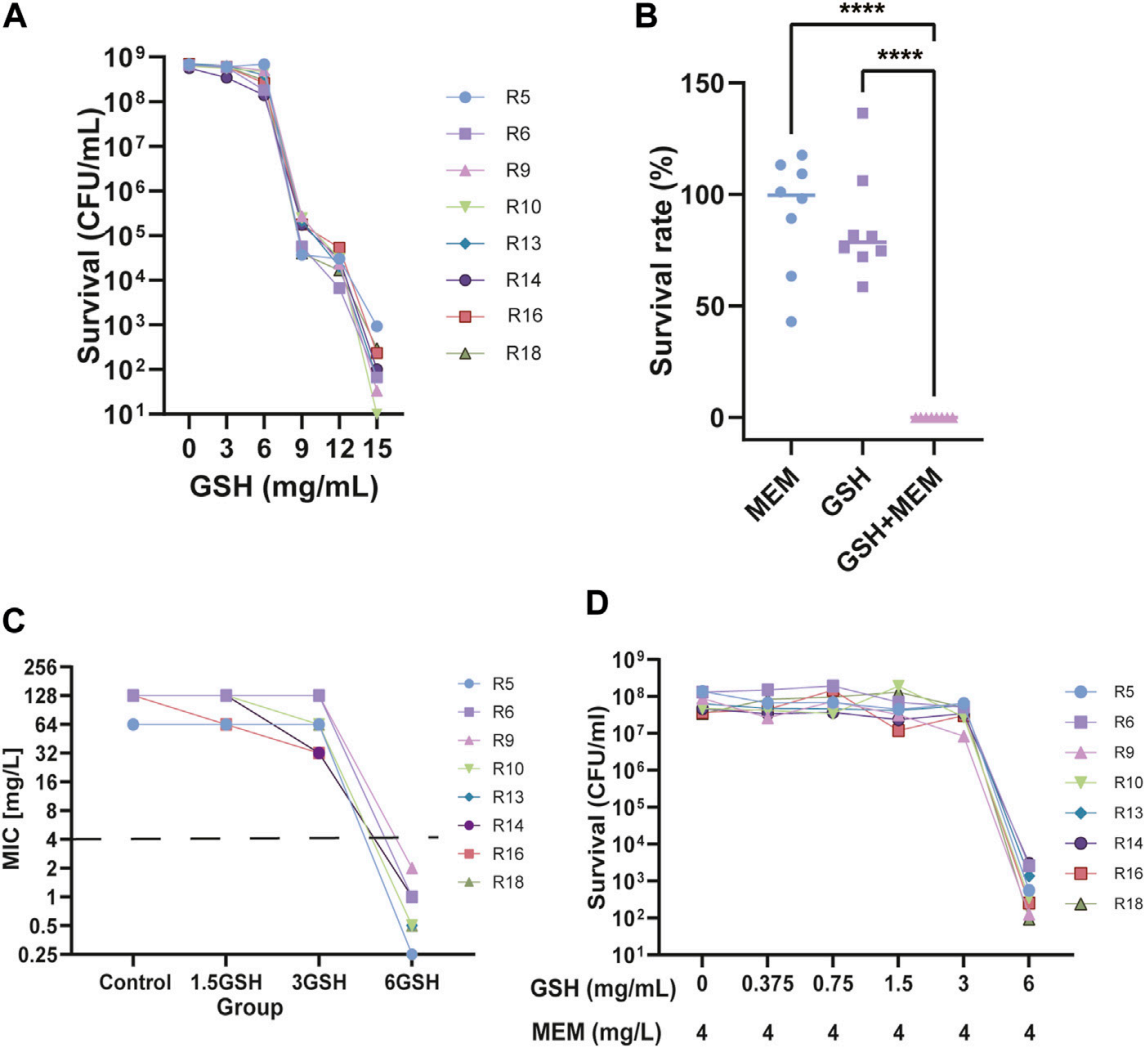
Effect of Glutathione (GSH) and meropenem on the growth of CRKP. **(A)** The number of surviving bacteria after using different concentrations of GSH for 24 h. **(B)** The survival rate of bacteria treatment with 4 mg/L meropenem (MEM), 6 mg/L GSH and in combination (GSH + MEM) after 24 h ****p < 0.0001. **(C)** The MIC of meropenem after adding different concentrations of GSH (1.5, 3, and 6 mg/mL). **(D)** The number of surviving bacteria after the use of 4 mg/L meropenem and different concentrations of GSH (0–6 mg/mL) for 24 h.

Most of the 30 isolates exhibited an MIC of 12 mg/mL for GSH (86.7%, Table 1). To precisely delineate the antibacterial efficacy of GSH, 8 strains were randomly selected, and the survival of clones in different concentrations of GSH were measured. Then, the minimum bactericidal concentration (MBC) was recorded. The results revealed that the isolates were not significantly affected by GSH in the range of 0–6 mg/mL (Figure 1A). However, as the concentration escalated from 6 to 9 mg/mL, there was a significant diminution in the surviving colony count. At 9 mg/mL, GSH effectively eradicated 99.9% of the bacteria, indicating an MBC of 9 mg/mL. Importantly, further augmentation of the GSH concentration led to a continued decrease in the bacterial survival rate. These results suggested that exogenous GSH inhibits CRKP growth.

To determine whether there was synergy between GSH and meropenem, the FICI for GSH in combination with meropenem was measured. Among the majority of clinical isolates, specifically 86.7% of isolates, the FICI for the combination of GSH and meropenem approached 0.5, denoting an additive effect (Table 3). Conversely, the time-killing assay indicated that GSH or meropenem alone could not inhibit bacterial growth, while the combination of GSH and meropenem significantly antagonized the bacteria after 24 h of exposure, denoting a synergistic effect (Figure 1B). These data together suggest that GSH is synergistic with meropenem *in vitro* to fight against infections by CRKP.

**TABLE 3:** Fractional inhibitory concentration (FIC) indices for GSH in combination with meropenem.

FIC index	Isolate	# of isolate
0.5	R30	1
0.503	R6, R13, R16, R23	4
0.507	R2, R4, R5, R7, R10, R11, R12, R14, R17, R19, R20, R21, R22, R25, R29	15
0.515	R9, R15, R18, R24, R26, R28	6
0.75	R1, R8	2
1	R3, R27	2
		Total: 30

### 3.3 Exogenous GSH prominently reverses meropenem resistance in CRKP

**FIGURE 2:**
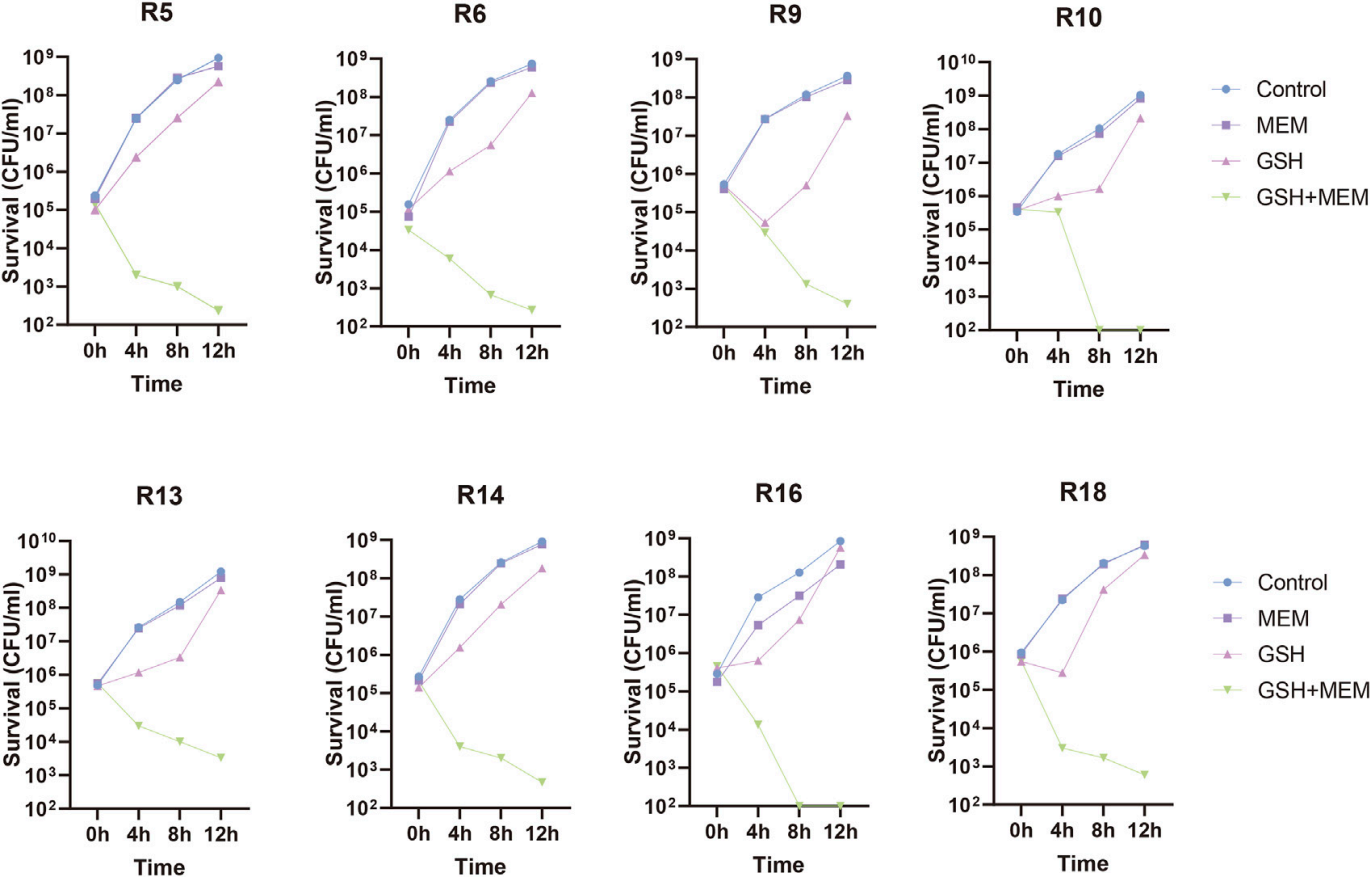
Time-killing curves of 8 CRKP treated with 4 mg/L meropenem (MEM), 6 mg/L GSH and in combination (GSH + MEM). Bacteria growing in blank culture medium as control group.

To further evaluate the potentiation, the effects of different concentrations of GSH on the sensitivity of meropenem were determined in these clinical isolates. The susceptibility of isolates to meropenem increased when the concentration of GSH was either 1.5 mg/mL or 3 mg/mL, with the maximum improvement reaching up to a 4-fold increase in susceptibility (Figure 1C). Nevertheless, not all isolates demonstrated augmented sensitivity at these particular concentrations. Importantly, the susceptibility of all isolates to meropenem significantly increased (≥32-fold) upon the introduction of 6 mg/mL GSH. This result implies that the potentiation of meropenem sensitivity by GSH was contingent on concentration. To further validate this result, the number of viable bacteria exposed to a 2-fold gradient concentration of GSH combined with 4 mg/L meropenem for 18–24 h was recorded. The results indicated that when combined with 4 mg/L meropenem, neither 1.5 mg/mL nor 3 mg/mL GSH affected the survival rate of bacteria (Figure 1D). Only when the GSH concentration was elevated to 6 mg/mL were the isolates growth significantly suppressed (≥99.9%). The decrease in viable bacteria occurred as early as 4 h after treatment and persisted through 24 h with the combination of 6 mg/mL GSH and 4 mg/L meropenem (Figure 1B; Figure 2).

### 3.4 Exogenous GSH stimulates excess ROS production in CRKP

**FIGURE 3:**
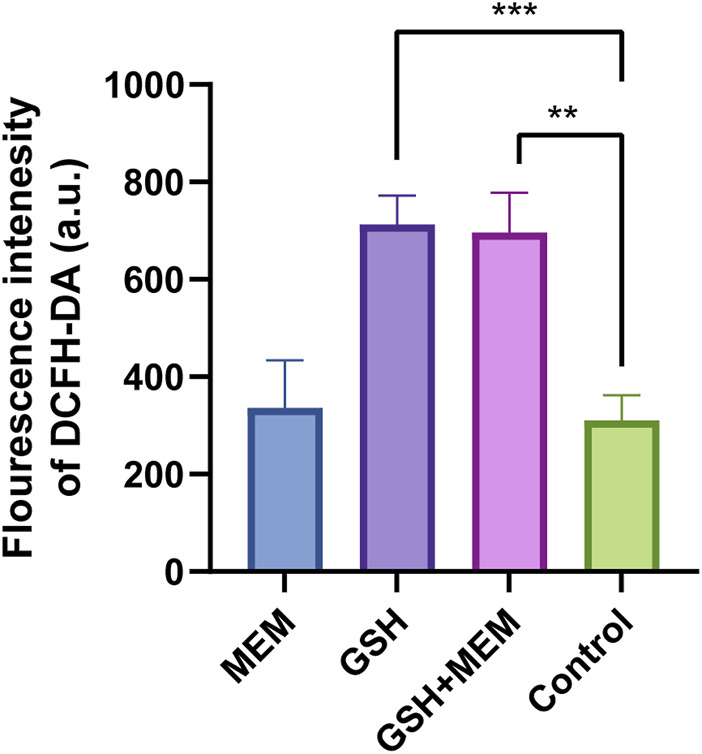
Fluorescence intensity of DCFH-DA in each group. MEM, meropenem 4 mg/L; GSH, glutathione 6 mg/mL; GSH + MEM, meropenem 4 mg/L and glutathione 6 mg/mL; Control, untreated. **p < 0.01; ***p < 0.001.

Recent studies have suggested that oxidative stress due to the increase in ROS caused by antibiotics is a common antimicrobial mechanism (Van Acker and Coenye, 2017; Hong et al., 2019). GSH is an antioxidant that helps maintain the optimum intracellular redox (Masip et al., 2006). The addition of exogenous GSH may cause the original redox homeostasis to be disrupted and increase the production of ROS, thus promoting sterilization. Therefore, we hypothesized that the antimicrobial effects of GSH might result from the overproduction of intracellular ROS. The results indicated that compared to the control group or the group treated with meropenem alone, the bacteria treated with GSH exhibited a significant increase in intracellular ROS levels after a 2 h treatment (Figure 3). Moreover, the combination GSH and meropenem treatment significantly increased the intracellular ROS levels. This result was consistent with the hypothesis that the addition of exogenous GSH promoted the production of ROS in *K. pneumoniae*. However, although there was a significant difference in bacterial survival between the GSH group and the combination group (Figure 1B), there was no significant difference in ROS production between the two groups, indicating that the bactericidal effect of the GSH-meropenem combination therapy was unlikely to be associated with the increase of ROS production.

### 3.5 The combination of GSH and meropenem led to significant metabolic disruptions in CRKP

**FIGURE 4:**
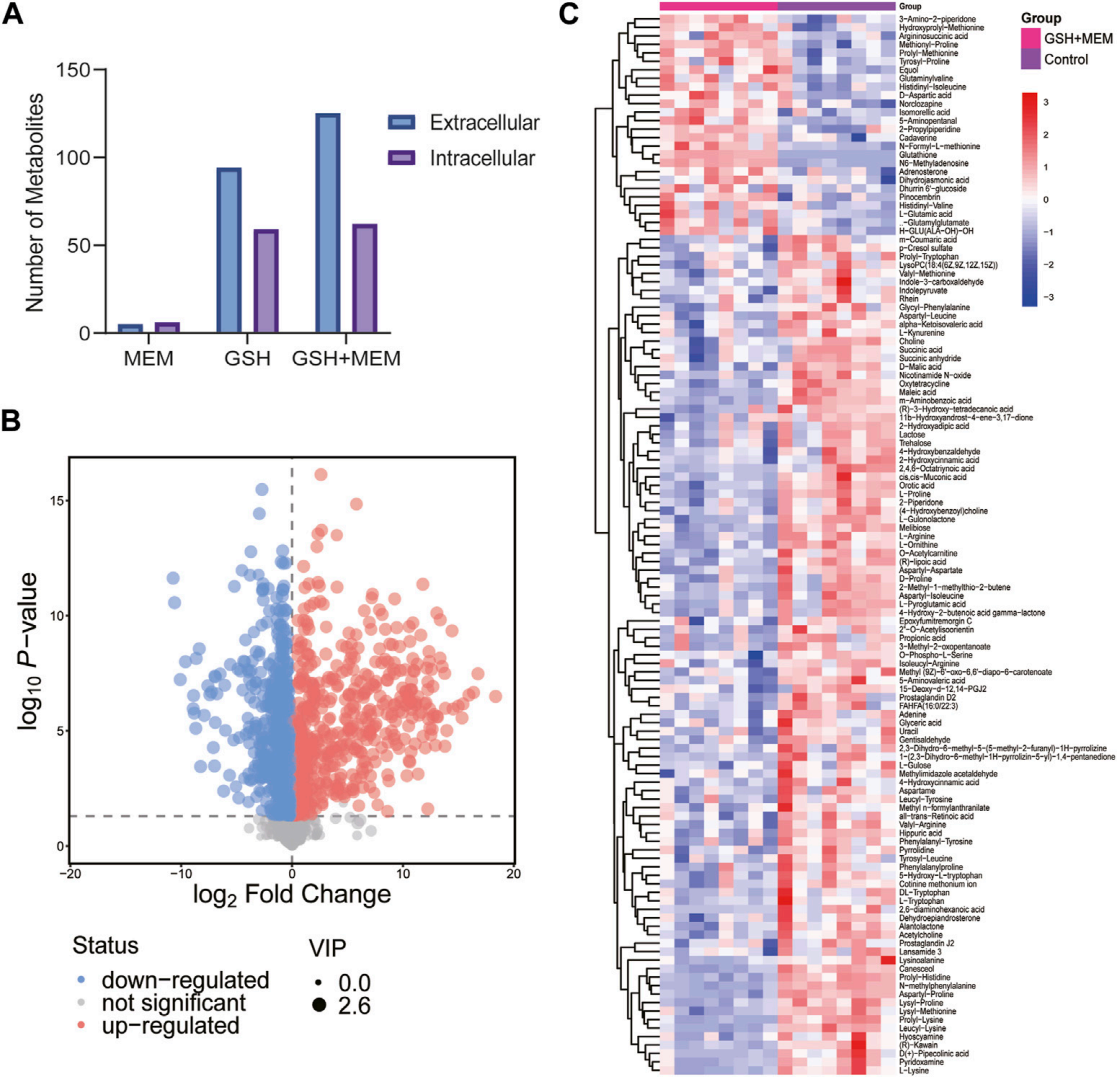
Metabolomics analysis results. **(A)** Number of different changed metabolites in each group. MEM, 4 mg/L meropenem; GSH, 6 mg/L glutathione; GSH + MEM, 6 mg/L glutathione and 4 mg/L meropenem. **(B)** Volcano plot of extracellular metabolites for the combination group vs. control group. **(C)** Clustered heat map profiles of the relative abundance for significantly affected extracellular metabolites in CRKP. Blue = significant decrease, red = significant increase. The metabolites with VIP>1 and *p* < 0.05 were considered as significantly changed metabolites.

The resistance mechanisms of bacteria are associated with their metabolic state (Kok et al., 2022). To explore the potential mechanism by which GSH increases the sensitivity of CRKP to meropenem, the metabolic characteristics of strains were assessed. Strains in exponential growth were collected by centrifugation and resuspended in medium with a single drug or two drugs for 4 h, and strains resuspended in blank medium were used as controls. Eight biological replicates were used for each group. A total of 500 and 573 putatively identified metabolites were obtained in the extracellular and intracellular metabolomes, respectively (Supplementary Table 1 and 2). PCA was carried out to assess the quality of samples. All samples were located within Hotelling’s T-squared (95%) ellipse, and all QC samples were clustered together in the PCA score scatter plot (Supplementary Figure 1). OPLS-DA was applied to visualize group separation and identify metabolites with significantly different abundances. The GSH, meropenem or the combination group were clearly distinguished from the control group in the OPLS-DA score plot (Supplementary Figure 2). Significantly differential metabolites were screened, and the corresponding VIP and *p*-values for each metabolite can be found in Supplementary Materials 2 and 3. Meropenem alone induced minor metabolic changes, while GSH alone significantly changed the abundance of 94 and 59 extracellular and intracellular metabolites, respectively (Figure 4A). However, the results revealed that a total of 125 and 62 metabolites in the external and internal metabolome, respectively, had significant variations in the combination group (Figure 4). These results indicated that the antibacterial effect of the combination of GSH and meropenem may be due to profound changes in bacterial metabolism. Compared to the individual use of GSH, the combination with meropenem resulted in a greater number of differential metabolites in the external metabolome. Thus, the 125 significantly differential metabolites in the external metabolome were mapped to the KEGG database for further analysis to identify the most affected pathways.

### 3.6 KEGG pathway analysis of the significantly differential metabolites

**FIGURE 5:**
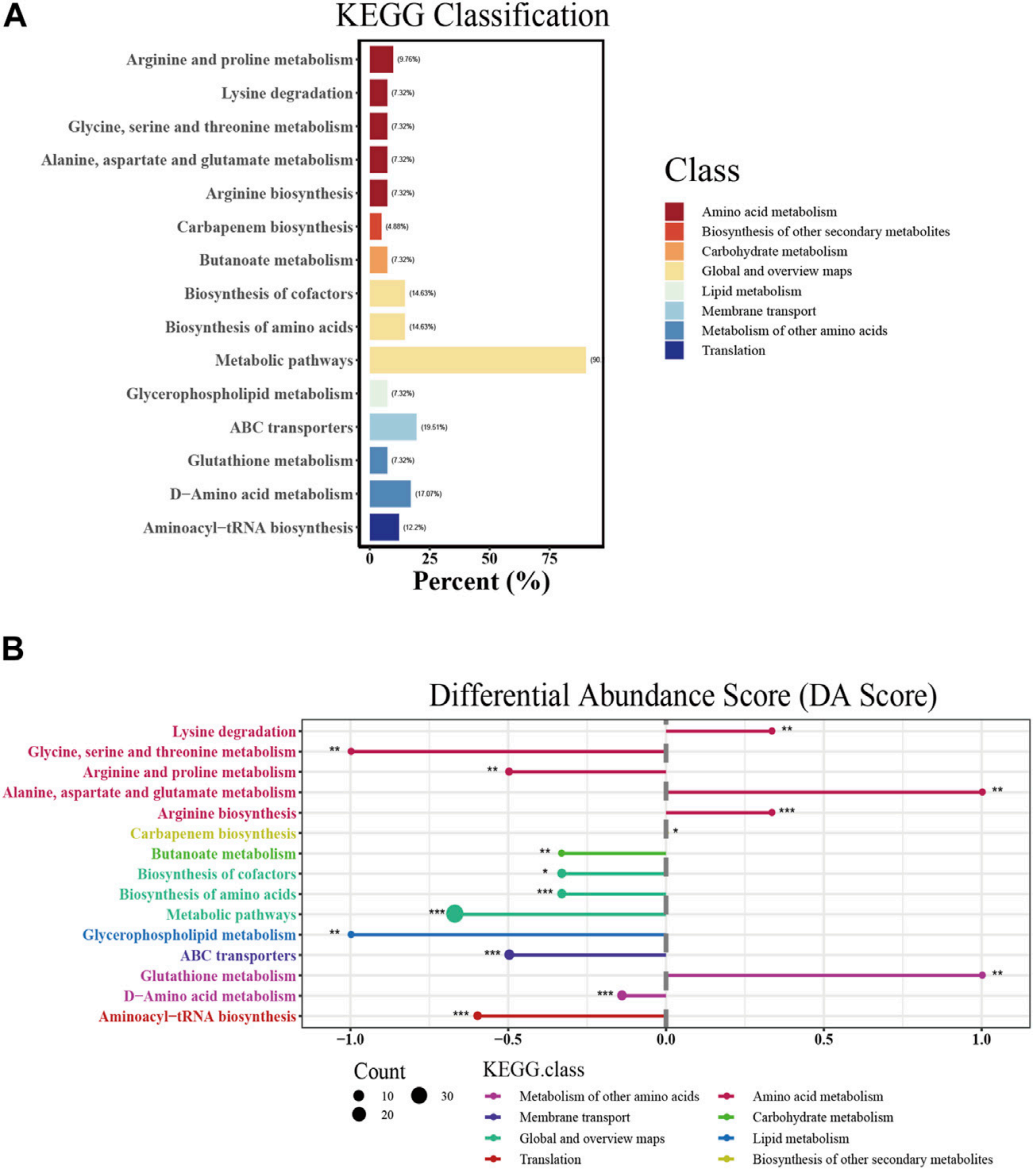
KEGG pathway analysis of significantly affected extracellular metabolites. **(A)** KEGG classification of each pathway. The abscissa represents the percentage of annotated metabolites in a certain pathway compared to all annotated metabolites. **(B)** Differential abundance score (DA score) of each pathway. The size of the dot indicates the number of significantly affected metabolites annotated in the pathway. DA score reflects the overall changes of all metabolites in the metabolic pathway. A score of 1 indicates an upward trend in the expression of all annotated metabolites in the pathway, while −1 indicates a downward trend in the expression of all annotated metabolites in the pathway. The length of the line segment represents the absolute value of DA score.

According to the annotation results, the significantly differential metabolites were mainly involved in amino acid metabolism, carbohydrate metabolism, lipid metabolism, membrane transport and translation (Figure 5A). Then, the differential abundance scores (DA scores) were calculated, revealing that glycerophospholipid metabolism; glycine, serine and threonine metabolism; arginine and proline metabolism; ABC transporters; aminoacyl-tRNA biosynthesis; D-amino acid metabolism; and butanoate metabolism in the combination group were significantly inhibited (Figure 5B). In contrast, alanine, aspartate and glutamate metabolism, arginine biosynthesis and lysine degradation were promoted. KEGG analysis revealed the involvement of the differentially abundant metabolites in a spectrum of metabolic pathways, encompassing multiple amino acid metabolism pathways, membrane transport pathways and translation. More importantly, their substantial influence on glycerophospholipid metabolism, which was related to antibiotic sterilization, was noted (Dalebroux, 2017; Dias et al., 2018).

### 3.7 GSH disrupted glycerophospholipid metabolism and increased membrane permeability in CRKP

**FIGURE 6:**
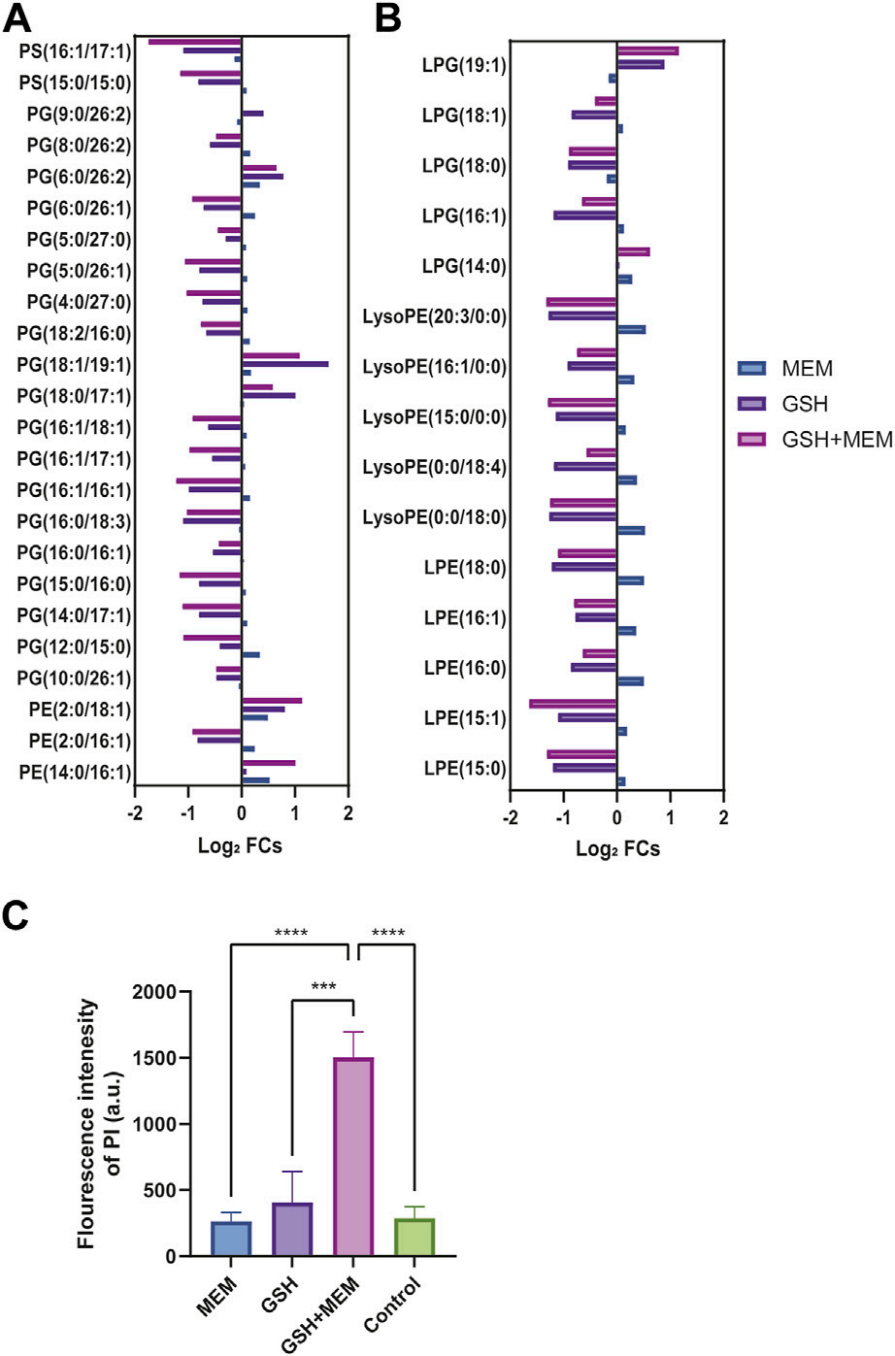
Glycerophospholipid metabolism and membrane permeability. **(A)** The log_2_ fold change of glycerophospholipids in each group. **(B)** The log_2_ fold change of lysophospholipids in each group. **(C)** The fluorescence intensity of PI in each group. Control, untreated; MEM, 4 mg/L meropenem; GSH, 6 mg/L glutathione; GSH + MEM, 6 mg/L glutathione and 4 mg/L meropenem. ***p < 0.001; ****p < 0.0001.

Glycerophospholipids constitute vital elements within the dual-membrane envelope of Gram-negative bacteria (Dalebroux, 2017). In this study, GSH and meropenem significantly interfered with the metabolism of glycerophospholipids (Figure 5B); thus, we performed a further analysis of the intracellular metabolome. In the internal metabolome, two types of phosphatidylserines (PSs) were identified. In both the GSH monotherapy and combined meropenem treatment groups, these two PSs exhibited significant reductions, while there were no substantial changes observed in the meropenem monotherapy group (Figure 6A). Phosphatidylethanolamine (PE) is the most abundant membrane phospholipid within prokaryotic cells, which originates from the decarboxylation of PS (Vance and Steenbergen, 2005). Noteworthy differences were observed in three PEs in this study. Notably, PE (2:0/16:1) exhibited a marked reduction in both the GSH and combination groups. Conversely, within the combination group, both PE (2:0/18:1) and PE (14:0/16:1) were substantially elevated. Meanwhile, a PE (2:0/18:1) increase was only discernible in the GSH group (Figure 6A). The decrease in PS and the increase in PE were more notable in the combination group than in the GSH group. The aminoacylation of phosphatidylglycerol (PG) constitutes a crucial mechanism that is employed by bacteria to withstand environmental stress (Slavetinsky et al., 2017). The results revealed a significant reduction in various PGs in both the GSH and combination groups. Particularly, the levels of PG (18:1/19:1) and PG (18:0/17:1) were reduced. Intriguingly, the GSH group exhibited a marginally greater increase in PG levels when compared to the combination group, which is different from the change in PS and PE.

Lysophospholipids, which usually make a contribution of less than 1% to the formation of the bacterial envelope, can also be converted into PG, PE, and cardiolipin (Zheng et al., 2017). The results clearly demonstrated the contrasting effects of GSH or combined treatment *versus* meropenem on lysophosphatidylethanolamine (LPE) and lysophosphatidylglycerol (LPG). GSH and combined treatment primarily reduced their levels, while meropenem led to a slight increase (Figure 6B).

When considering that GSH combination with meropenem could disrupt glycerophospholipid metabolism, we further assessed its impact on the membrane permeability in CRKP. Consistent with previous studies, the fluorescence intensity of PI was used to label the membrane permeability of CRKP (Song et al., 2020). The results clearly showed that enhanced membrane permeability occurred solely when GSH was employed in conjunction with meropenem, whereas there was no such change when either GSH or meropenem was administered individually (Figure 6C).

## 4 Discussion

This study demonstrated that exogenous GSH had a synergistic effect with meropenem *in vitro*. Interestingly, this change mainly resulted from the increased sensitivity to meropenem rather than GSH activity. In addition, this study provides some indications of the underlying mechanisms. Interestingly, the metabolomics results showed that the synergistic effect was related to metabolic disorders, especially the substantial influence on glycerophospholipid metabolism, which significantly increased membrane permeability.

It has been reported that the mortality rate of MDR *K. pneumoniae* infection is higher than 40%–50% and these infections represent a major challenge in the field of infectious disease treatment (Xu et al., 2017). Appropriate treatment is essential for controlling MDR *K. pneumoniae* infection, especially in critically ill patients (Seo et al., 2020). However, due to the emergence of CRKP, treatment options are limited, so the choice of appropriate treatment has become a problem. Antibiotic combination therapy has been the most widely used during recent years. Despite resistance to carbapenem, some studies confirmed the effectiveness of meropenem-based regimens in the treatment of these infections (Daikos et al., 2014). Interestingly, some studies have revealed that the combination of non-antibiotic drugs and antibiotics that could confront MDR bacteria (Liu et al., 2019; Liu et al., 2021). Previous studies have shown that GSH alone mediated antibacterial effectiveness in *E. coli*, *Pseudomonas aeruginosa* and *Acinetobacter baumannii* with a dose-dependent manner (Goswami and Jawali, 2007; Klare et al., 2016; Alharbe et al., 2017; Das et al., 2019). Similar to previous studies, our research indicated that GSH has a bactericidal effect in *K. pneumoniae*. Furthermore, GSH and meropenem exhibited a synergistic effect. However, this study mainly focused on CRKP, the effect of the GSH-meropenem combination should be evaluated in a broader range of antibiotic-resistant bacteria. This result was similar to that of Goswami et al., which shows that GSH can be used as a synergist with ampicillin and penicillin (Goswami and Jawali, 2007). Additionally, we evaluated the ability of GSH to promote the sensitivity to meropenem. Surprisingly, at 6 mg/mL, GSH can reduce the MIC of meropenem to 2 mg/L, although the initial MIC of meropenem was greater than 64 mg/L. When 6 mg/mL GSH was used in combination with 4 mg/L meropenem, it can kill bacteria as early as 8 h after treatment. Previous studies have shown that 30 mM GSH could act as a biofilm disruptor in many bacterial species (Das et al., 2019). In our study, the effective concentration of GSH for the synergistic and sensitization effects was 6 mg/mL (20 mM), and the effective concentration when used alone was between 6 and 12 mg/mL (20–40 mM), similar to previous results.

GSH systems are a major thiol-dependent systems that provide promising antioxidant capacity targets for novel antibiotic development (Song et al., 2021). Recent studies have revealed that ebselen, auranofin and combining ebselen and silver exhibited a strong synergistic effect against MDR Gram-negative bacteria (Ren et al., 2020). The addition of exogenous GSH may disrupt the balance of the GSH system. GSH metabolism was analyzed in this study based on metabolic results and total ROS. The metabolic results of GSH alone or in combination with meropenem showed significant changes in the GSH metabolism pathway, and the total intracellular ROS increased. The change in the GSH pathway was consistent with the increase in ROS. However, the lack of a significant difference in ROS between the combination group and the GSH monotherapy group indicated that ROS may not have a significant correlation with this synergistic effect, and the specific mechanism needs further research. This results were similar to research on GSH-mediated augmentation of ampicillin and penicillin antibacterial activity against *E. coli* that showed that this effect was independent of its redox status outside the cell and the status of gamma glutamyl-transepeptidase (Goswami and Jawali, 2007).

Glycerophospholipid alterations can result from bacterial resistance and can also serve as the fundamental mechanism contributing to the synergy of drugs (Aye et al., 2020; Song et al., 2020). Song et al. reported that SLAP-S25 reverses the resistance of MDR Gram-negative pathogens by binding to both lipopolysaccharide and PG and triggering membrane damage to potentiate antibiotic efficacy (Song et al., 2020). In this study, we observed that the combined use of GSH and meropenem intensified the alterations in glycerophospholipid levels. This was confirmed by the increase in cell membrane permeability. Bacterial membrane damage has been reported boosting the action of antibiotics (Cai et al., 2023). These results suggest that the mechanism of synergy may involve multiple changes in glycerophospholipid levels, which led to an increase in membrane permeability. However, the specific mechanism needs further research. In addition, GSH in combination with meropenem also impacted the metabolism of multiple amino acids, which is related to the synthetic peptidoglycan skeleton (Tsai et al., 2016; Antonoplis et al., 2019; Charlier and Bervoets, 2019; Pereira et al., 2020; Miyamoto and Homma, 2021), indicating that the sensitizing effect of GSH on meropenem may be related to the inhibition of cell wall synthesis.

Although we investigated the synergistic effects of GSH and meropenem against CRKP *in vitro*, this study has certain limitations. One limitation is that further *in vivo* experiments are still needed. In addition, while the findings are promising, clinical validation in real-world patient populations is necessary to confirm the effectiveness and safety of the GSH-meropenem combination. However, we hope that future work will show that GSH-meropenem combination is a promising treatment candidate to improve the treatment of drug-resistant bacteria in the clinic.

## 5 Conclusion

In this study, the antibacterial effect of GSH was verified in CRKP isolated from the clinic, and the sensitizing effect of GSH on meropenem was verified. The possible mechanism of this sensitization was the increase in membrane permeability due to alterations to glycerophospholipids. The above results provide a new direction for the treatment of CRKP.

## Data Availability

The original contributions presented in the study are included in the article/Supplementary Material, further inquiries can be directed to the corresponding author.
