# Supplementary material for: Exogenous glutathione reverses meropenem resistance in carbapenem-resistant Klebsiella pneumoniae
Source: Front Pharmacol. 2023 Dec 19;14:1327230. doi: 10.3389/fphar.2023.1327230 (PMC10762803; doi:10.3389/fphar.2023.1327230)
Supplement: Supplementary file 1 [file DataSheet4.docx]

Supplementary Material

# Supplementary Tables (Supplementary Material 1)

**Supplementary Table 1.** A total of 500 putatively identified metabolites obtained in the extracellular metabolomes.

**Supplementary Table 2.** A total of 573 putatively identified metabolites obtained in the intracellular metabolomes.

# Supplementary Figure

**Supplementary Figure 1.** The PCA score scatter plot of each group. Control, untreated; GSH, 6mg/ml glutathione; MEM, 4mg/L meropenem; GSH+MEM, 6mg/ml GSH+4mg/L meropenem; QC, quality control.


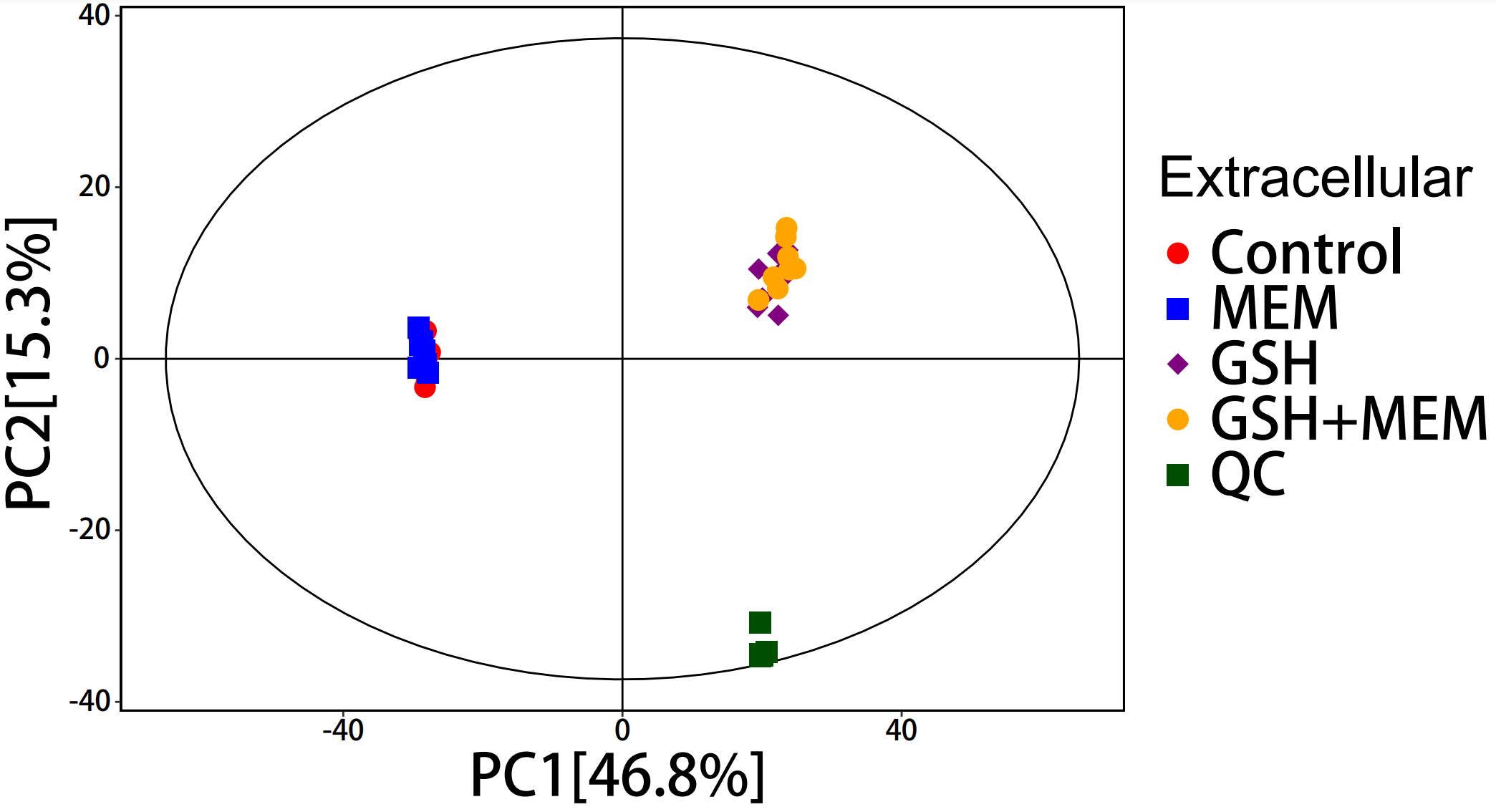


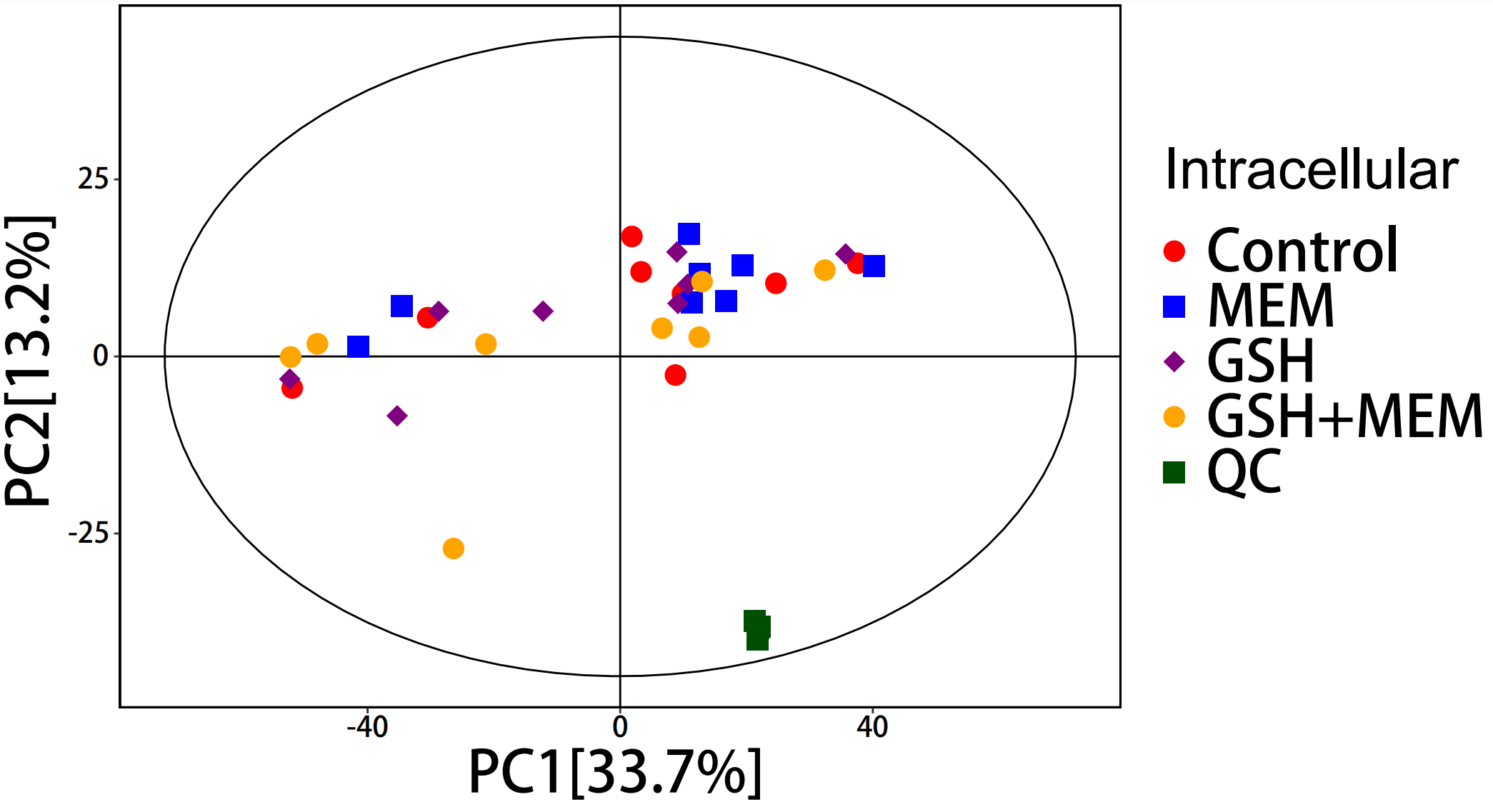


**Supplementary Figure 2.** The OPLS-DA score scatter plot of each group. Control, untreated; GSH, 6mg/ml glutathione; MEM, 4mg/L meropenem; GSH+MEM, 6mg/ml GSH+4mg/L meropenem.

**
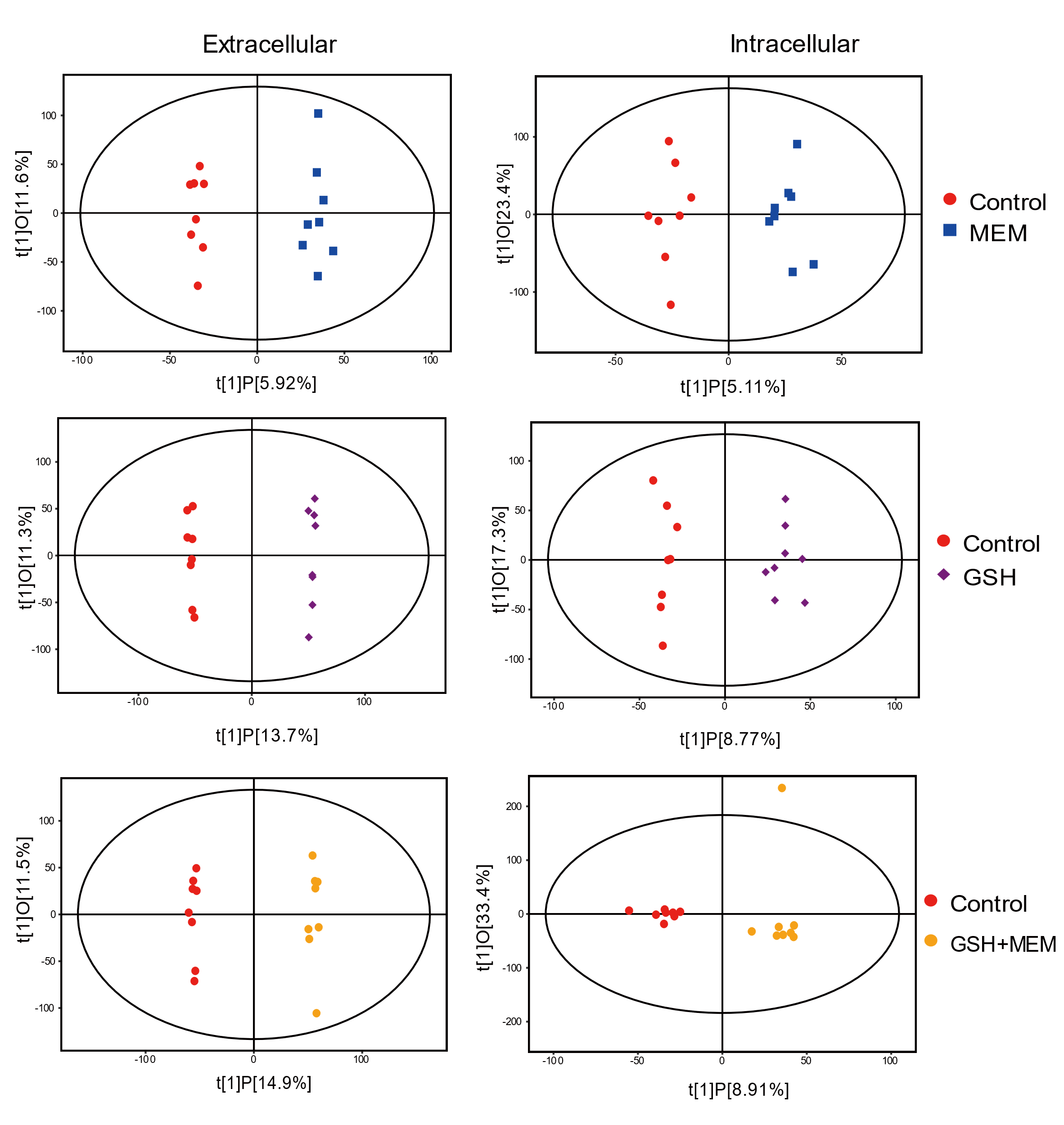
**

# Supplementary Data

**Supplementary material 2**. Extracellular significantly different metabolites.

**Supplementary material 3.** Intracellular significantly different metabolites.
